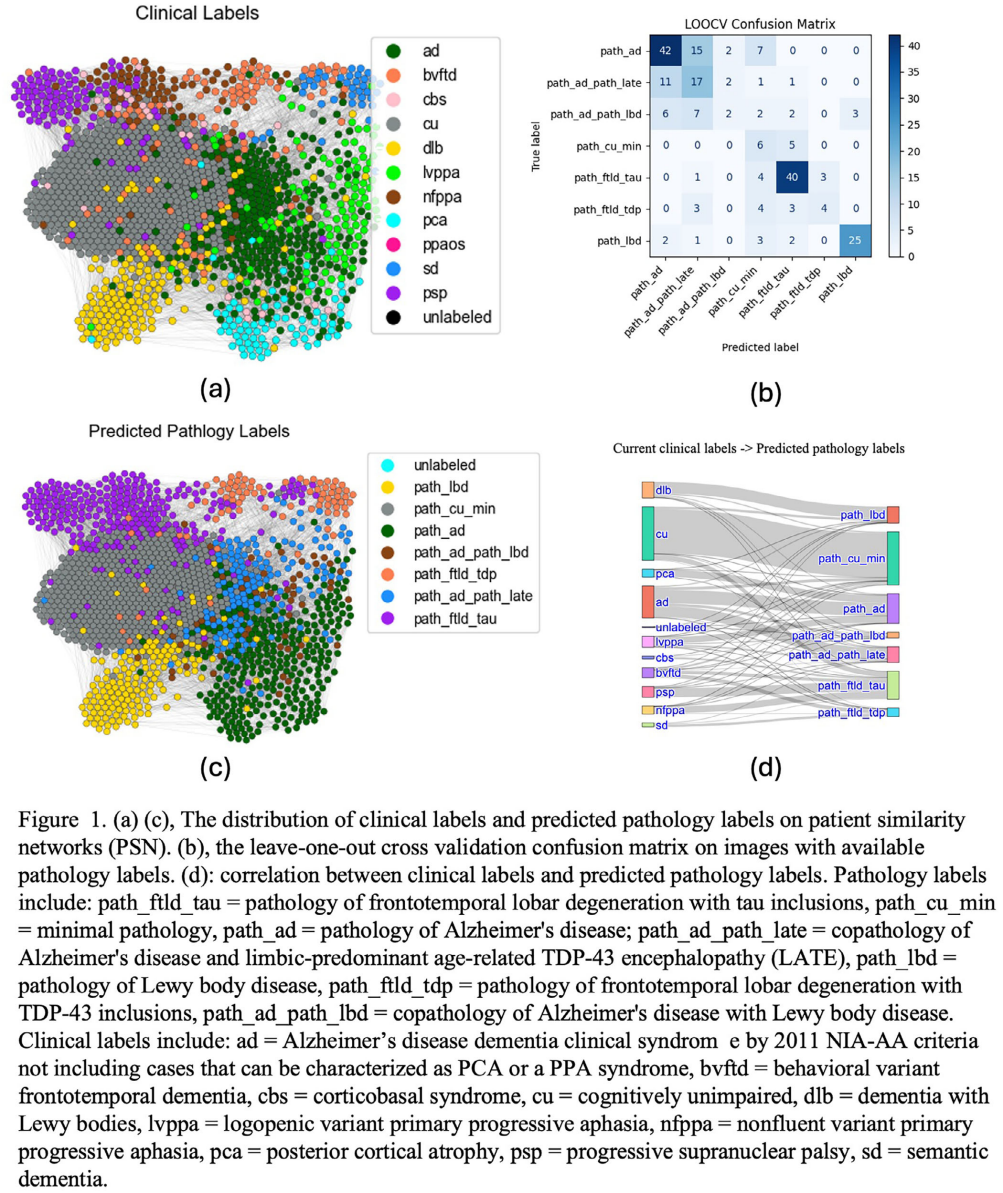# Prediction of neuropathology using the FDG‐PET based patient similarity network

**DOI:** 10.1002/alz70855_103746

**Published:** 2025-12-24

**Authors:** Gemeng Zhang, Leland Barnard, Hugo Botha, Nick Corriveau‐Lecavalier, Venkatsampath Gogineni, Jonathan Graff‐Radford, Brian J Burkett, Derek R. Johnson, Sean J. Huls, Aditya Khurana, John L. Stricker, Hoon‐Ki Min, Matthew L. Senjem, Winnie Z Fan, Heather J. Wiste, Mary M. Machulda, Melissa E. Murray, Dennis W. Dickson, Aivi T. Nguyen, R. Ross Reichard, Jeffrey L. Gunter, Christopher G Schwarz, Kejal Kantarci, Jennifer L. Whitwell, Keith A. Josephs, David S. Knopman, Brad F Boeve, Ronald Petersen, Clifford R. Jack, Val J Lowe, David T. Jones

**Affiliations:** ^1^ Department of Neurology, Mayo Clinic, Rochester, MN, USA; ^2^ Department of Psychiatry and Psychology, Mayo Clinic, Rochester, MN, USA; ^3^ Department of Radiology, Mayo Clinic, Rochester, MN, USA; ^4^ Department of Information Technology, Mayo Clinic, Rochester, MN, USA; ^5^ Department of Health Sciences Research, Mayo Clinic, Rochester, MN, USA; ^6^ Department of Neuroscience, Mayo Clinic Florida, Jacksonville, FL, USA; ^7^ Mayo Clinic, Jacksonville, FL, USA; ^8^ Department of Laboratory Medicine and Pathology, Mayo Clinic, Rochester, MN, USA

## Abstract

**Background:**

Neuropathologic evaluation is essential for diagnosing and treating dementia, but definitive diagnoses is only available after post‐mortem brain autopsy. Positron emission tomography (PET) offers a potential in vivo proxy for predicting neuropathology. This study proposes a semi‐supervised learning (SSL) model using a single FDG‐PET image to predict underlying neuropathology.

**Method:**

The SSL model leverages a self‐organized graph structure based on a patient similarity network (PSN) created from 1,495 FDG‐PET images across the aging and neurodegenerative spectrum. Each patient is represented by a node, with edges indicating image similarity derived from principal component analysis features and cosine similarity weights. The PSN structure connects 20 nearest neighbors. Neuropathologic labels were available for 204 autopsied patients.

The Poisson Learning Method was employed to propagate labels from these annotated nodes to unlabeled nodes, guided by the PSN structure. FDG‐PET images were collected from clinical and research participants at the Mayo Clinic and processed using an MRI‐free pipeline. Neuropathologic labels were assigned based on widely accepted diagnostic criteria. Leave‐one‐out cross‐validation (LOOCV) was used to assess model performance.

**Results:**

The confusion matrix demonstrated high accuracy in predicting Alzheimer's disease, frontotemporal lobar degeneration with tau inclusions, and Lewy body disease pathology. A Sankey diagram and label distribution analysis revealed strong correlations between clinical and predicted neuropathologic labels.

**Conclusion:**

This study highlights the potential of a graph‐based SSL pipeline to predict neuropathological changes using FDG‐PET imaging data, despite limited pathology labels. The findings underscore the value of SSL in bridging the gap between abundant neuroimaging data and scarce neuropathologic labels, contributing to improved dementia diagnosis and understanding.